# Use of fusion transcription factors to reprogram cellulase transcription and enable efficient cellulase production in *Trichoderma reesei*

**DOI:** 10.1186/s13068-019-1589-2

**Published:** 2019-10-15

**Authors:** Fangzhong Wang, Ruiqin Zhang, Lijuan Han, Wei Guo, Zhiqiang Du, Kangle Niu, Yucui Liu, Chunjiang Jia, Xu Fang

**Affiliations:** 1 State Key Laboratory of Microbial Technology, Shandong University, Qingdao, 266237 China; 2 Center for Biosafety Research and Strategy, Tianjin University, Tianjin, China; 3 School of Chemistry and Chemical Engineering, Shandong University, Jinan, Shandong China

**Keywords:** Fusion transcription factors, Carbon catabolite repression, Biomass, Cellulase, *Trichoderma reesei*, Cre1

## Abstract

**Background:**

*Trichoderma reesei* is widely used for cellulase production and accepted as an example for cellulase research. Cre1-mediated carbon catabolite repression (CCR) can significantly inhibit the transcription of cellulase genes during cellulase fermentation in *T*. *reesei*. Early efforts have been undertaken to modify Cre1 for the release of CCR; however, this approach leads to arrested hyphal growth and decreased biomass accumulation, which negatively affects cellulase production.

**Results:**

In this study, novel fusion transcription factors (fTFs) were designed to release or attenuate CCR inhibition in cellulase transcription, while Cre1 was left intact to maintain normal hyphal growth. Four designed fTFs were introduced into the *T*. *reesei* genome, which generated several transformants, named Kuace3, Kuclr2, Kuace2, and Kuxyr1. No obvious differences in growth were observed between the parent and transformant strains. However, the transcription levels of *cel7a*, a major cellulase gene, were significantly elevated in all the transformants, particularly in Kuace2 and Kuxyr1, when grown on lactose as a carbon source. This suggested that CCR inhibition was released or attenuated in the transformant strains. The growth of Kuace2 and Kuxyr1 was approximately equivalent to that of the parent strain in fed-batch fermentation process. However, we observed a 3.2- and 2.1-fold increase in the *p*NPCase titers of the Kuace2 and Kuxyr1 strains, respectively, compared with that of the parent strain. Moreover, we observed a 6.1- and 3.9-fold increase in the *p*NPCase titers of the Kuace2 and Kuxyr1 strains, respectively, compared with that of *Δcre1* strain.

**Conclusions:**

A new strategy based on fTFs was successfully established in *T*. *reesei* to improve cellulase titers without impairing fungal growth. This study will be valuable for lignocellulosic biorefining and for guiding the development of engineering strategies for producing other important biochemical compounds in fungal species.

## Background

Despite being the most abundant natural material, lignocellulose is a considerably under-utilized biomass [1, 2]. Lignocellulosic biorefining processes producing biofuels have attracted considerable attention in the past few decades owing to the global energy crisis caused by the extensive depletion of fossil fuels. In addition, large quantities of high-value chemical intermediates can be generated during the process [1–4]. One of the most important steps in lignocellulosic biorefining is the use of cellulases to release fermentable sugar from lignocellulose [5, 6]. However, a considerable limiting factor in industrial lignocellulosic biorefining is the high cost of the vast amounts of cellulases that are required for the degradation of lignocellulose (40- to 100-fold higher than that required for starch hydrolysis) [7–9].

One promising strategy for decreasing enzyme costs is to increase cellulase production by filamentous fungi [10]. However, the presence of d-glucose or d-xylose during cellulase fermentation triggers carbon catabolite repression (CCR), which significantly decreases cellulase transcription in filamentous fungi [11]. Therefore, the release or attenuation of CCR is a prerequisite for high-yield cellulase production [12–14]. In filamentous fungi, CCR is mediated by the conserved transcription factor Cre1. Long et al. [15] reported that the cellulase activity of *Δcre1* strain was 1.45-fold higher than that of its parent strain of *Trichoderma orientalis* EU7-22. Furthermore, Sun and Glass [16] demonstrated that the knockout of *cre1* in *Neurospora crassa* improved the expression of most cellulases and increased endocellulase activity by approximately 50%. Therefore, the deletion of *cre1* is widely accepted as an effective strategy for releasing CCR. However, several studies have revealed that *cre1* deletion impairs filamentous fungal growth, which leads to decreased final product titers. For example, the deletion of *cre1* in *T*. *reesei* or its replacement with a *cre1* mutant inhibited hyphal growth and affected final biomass accumulation, which led to decreased cellulase production [11, 17, 18]. In addition, *Δcre1* strain exhibited slower growth than its parent strain [16]. However, Rassinger et al. found that Cre1-96, a truncated from of Cre1, could bind to the cellulase promoter regions in *T*. *reesei* Rut-C30 and contributed to a partial release from CCR [19]. Furthermore, the constitutive expression of *cre1*-*96* could improve cellulase activity without impairing strain growth [20]. However, its deletion significantly decreased cellulase activity and resulted in serious growth defects [20]. These observations suggested that the truncation caused Cre1-96 to become an activator of cellulase expression rather than a repressor and that the functions of Cre1 exhibited a degree of plasticity. However, a structure-based approach for the rational design of Cre1 mutants is not feasible owing to the absence of crystal structure information.

The construction of synthetic transcription factors involves the fusion of transcription factor zinc finger domains with the activation domains of well-studied activators or repressors. They have demonstrated significant potential as a tool for creating phenotypic alterations in yeast and mammalian cells [21–24]. Replacement of the activation domain determines the specific role of a synthetic transcription factor [21]. Therefore, this strategy may provide a solution for the rational design of Cre1-based fusion transcription factors that can release or attenuate CCR in filamentous fungi. As previously mentioned, the deletion of *cre1* led to serious growth defects. Furthermore, Cre1 is required, albeit indirectly, for the positive regulation of key genes involved in fundamental life processes, such as energy biosynthesis and general metabolism [18]. Therefore, we proposed that the construction of fusion transcription factors based on Cre1 is an appropriate alternative for releasing or attenuating CCR in filamentous fungi. This would relieve the repressive effect of Cre1 on cellulase production; however, the maintenance of fungal growth will not be affected.

The industrial production of cellulase involves the fermentation of filamentous fungi, such as *T*. *reesei*, *N*. *crassa*, and *Penicillium oxalicum* [25–28]. Of these strains, *T*. *reesei* has the highest potential for the overproduction and secretion of cellulases [29, 30]. For example, the cellulase volumetric productivity rate of *T*. *reesei* CL847 can reach levels of 243 FPU/L/h in a 3000-L fermenter using lactose as a carbon source [31]. Furthermore, the cellulases currently required for global cellulosic ethanol production are predominantly derived from *T*. *reesei* [32].

The cellulase transcriptional regulatory network in *T*. *reesei* has been extensively explored in the last few years, and the functions of cellulase transcriptional repressors and activators in *T*. *reesei* have been comprehensively characterized [2]. Cre1 and Ace1 are cellulase transcriptional repressors that directly bind to the promoter regions of cellulase genes [2], and Ace3, Clr2, Ace2, and Xyr1 are transcriptional activators that enhance the transcriptional expression of cellulase genes [2, 33]. Therefore, we proposed that the *T*. *reesei* is a suitable system for screening novel synthetic transcription factors owing to their ability to release or attenuate CCR while retaining intact Cre1 to allow effective fungal growth. Such efforts will also accelerate the industrialization of biorefining processes.

Here, we proposed a novel strategy to alleviate CCR in *T*. *reesei* that was based on the construction of fusion transcription factors (fTFs). Four fTFs were constructed and integrated into the *T*. *reesei* genome and the phenotypic characteristics of the transformants were investigated in detail. Furthermore, we examined the transcription levels of the cellulase transcription factors and the major cellulase gene *cel7a* in the fTF transformants to confirm the effectiveness of our strategy for activating cellulase transcription. In addition, the cellulase titers of the transformants were compared with those of the parent and *Δcre1* strains using fed-batch culture in 3-L fermenters. This approach could also be employed in other fungal strains to release CCR while maintaining normal strain growth.

## Results

### Construction of fTFs

**Fig. 1:**
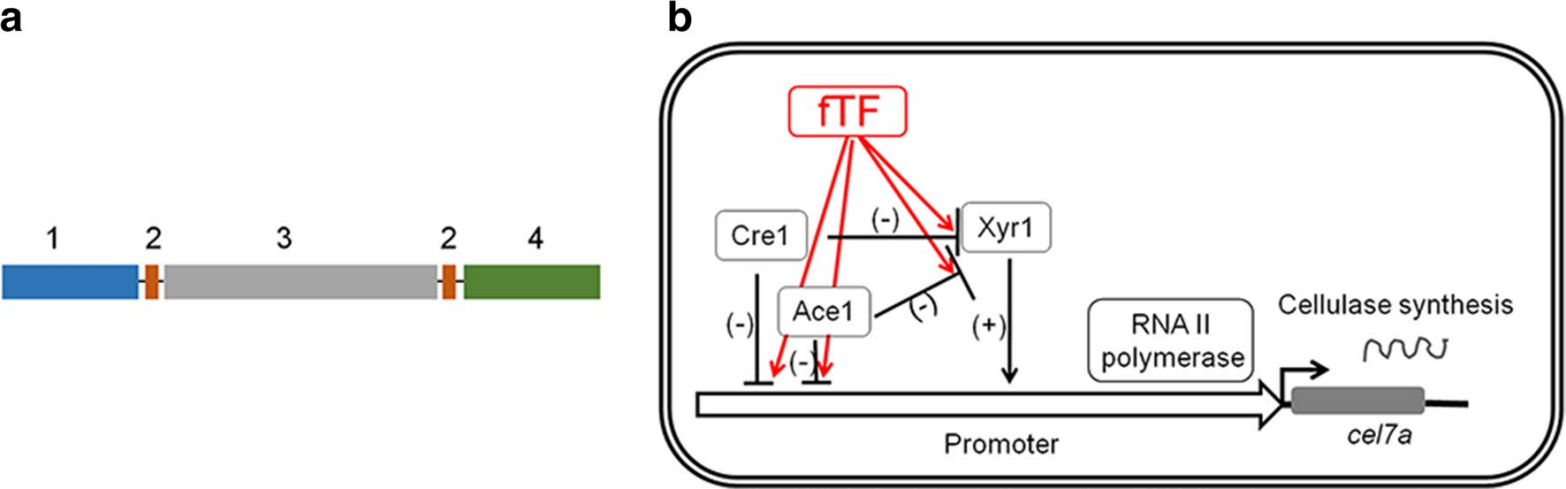
Schematic diagram and hypothetical working mechanism of fTFs in *T*. *reesei*. **a** 1: the Cre1 zinc finger region; 2: peptide linker: GSGGSGTS; 3: the activation domain of Ace3, Clr2, Ace2, or Xyr1; 4: the Ace1 zinc finger region. **b** − represents repression; + represents activation, and *cel7a* represents cellobiohydrolase 1

The fTFs were designed to include four parts: the activation domain of a cellulase activator protein (Ace3, Clr2, Ace2, or Xyr1), the zinc finger regions of Cre1 and Ace1, and a peptide used to link each domain (Fig. 1a). These fTFs were named *Sace3*, *Sclr2*, *Sace2*, and *Sxyr1.* Each fTF was under the control of the *T*. *reesei cre1* promoter and the *Aspergillus nidulans trpC* terminator (Additional file 1: Figure S1A). The zinc finger regions of the fTFs are believed to alleviate Cre1-and Ace1-mediated repression by competing for the Cre1- and Ace1-binding sites (Fig. 1b).

The constructed fTF cassettes were introduced into a *T. reesei Δtku70* strain to obtain the transformants, which were named Kuace3, Kuclr2, Kuace2, and Kuxyr1. Successful insertion of the cassettes was confirmed by Southern blotting (Additional file 1: Figure S1B, C). In addition, we constructed a control *T*. *reesei Δcre1* strain (Additional file 2: Figure S2).

### Phenotypic characterization of the transformants

**Fig. 2:**
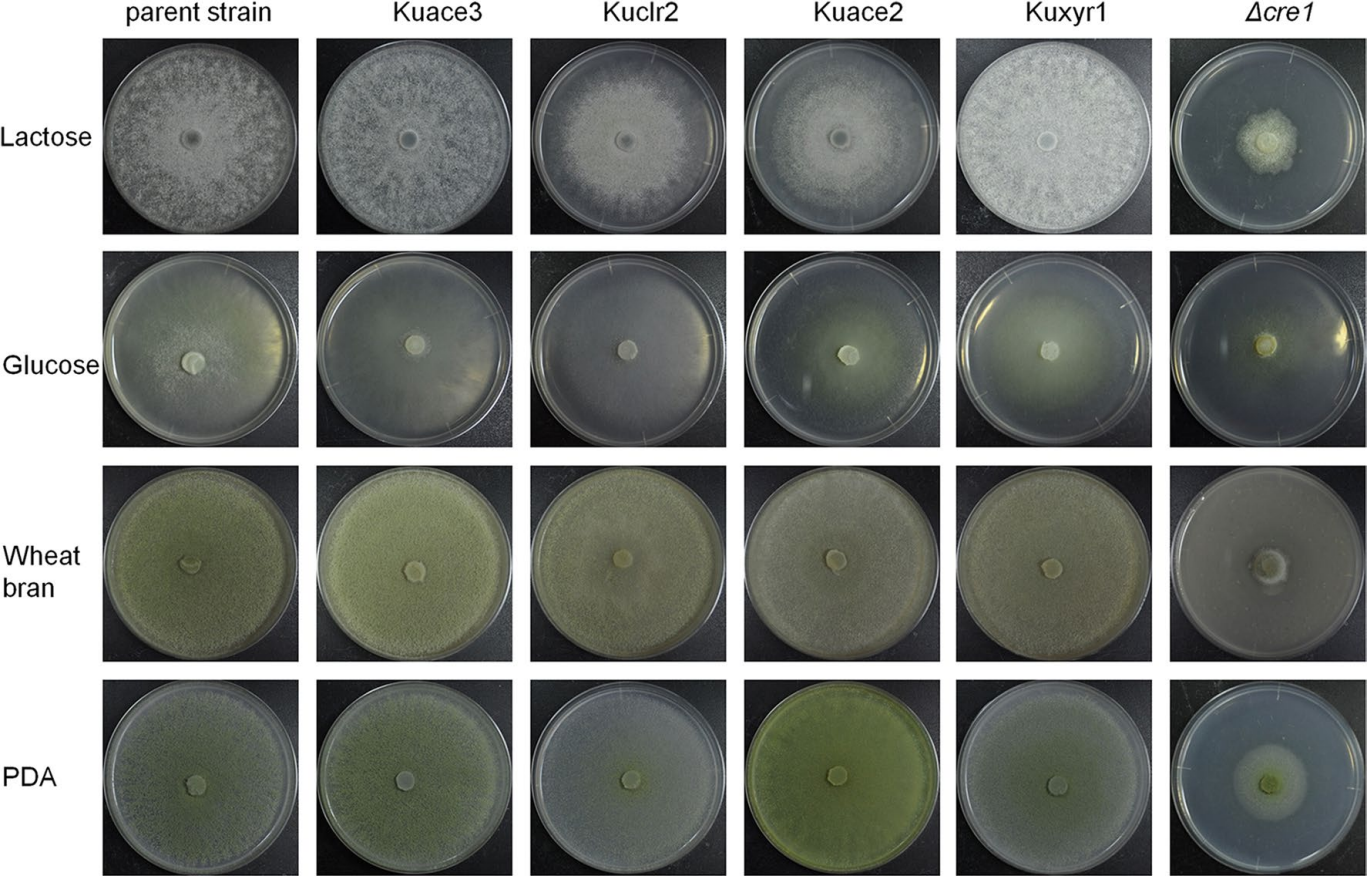
Morphologies of the parent strain and transformants on plates containing different carbon sources

Equivalent amounts of mycelia from the parent strain and transformants were inoculated on plates containing glucose, lactose, wheat bran, or potato dextrose agar (PDA). Phenotypic analysis of each strain was performed after incubating the plates for 6 days at 30 °C. Figure 2 shows the morphological changes observed in each strain on various plates. On plates containing lactose as a carbon source, the Kuclr2, Kuace2, and *T*. *reesei Δcre1* colonies became denser; in addition, the Kuace2 exhibited an annular distribution. Furthermore, significant differences in pigment production by the Kuclr2, Kuace2, *Δcre1*, and parent strains were observed on PDA and wheat bran-containing plates. On plates containing glucose, lactose, wheat bran, and PDA, the *T*. *reesei Δcre1* colonies were significantly smaller in diameter than those of the parent strain. In contrast, the colony diameters of Kuace3, Kuclr2, Kuace2, and Kuxyr1 were equivalent to those of the parent strain on PDA and wheat bran-containing plates (Additional file 3: Figure S3). We observed a slight decrease in the Kuclr2 and Kuace2 colony diameters when cultured on lactose-containing plates. Although the Kuace2 and Kuxyr1 colonies exhibited smaller diameters when cultured on plates containing glucose, their diameters were still higher than those of *T*. *reesei Δcre1*. Microscopic observation showed no hyphal differences among the Kuace3, Kuclr2, Kuace2, Kuxyr1, and parent strains. However, the hyphae of *T*. *reesei Δcre1* were notably shorter and thicker (Additional file 4: Figure S4). Next, the sporulation ability of all the strains was tested. The number of spores produced by all the transformants was found to drastically reduce. In particular, compared with the parent strain, the *Δcre1* strain produced five or sixfold lower numbers of spores on plates containing PDA or wheat bran, respectively (Additional file 5: Figure S5). Based on these observations, we concluded that the fTFs caused fewer morphological changes in *T*. *reesei* in comparison with *cre1* deletion. There were no apparent differences in hyphal growth between the parent and fTF-containing transformant strains, with the exception of Kuace2 and Kuxyr1 cultured on glucose-containing plates (Fig. 2 and Additional file 3: Figure S3).

### Transcriptional levels of *xyr1* and *cel7a* following the homologous integration of fTFs into the *T*. *reesei* genome

The growth of Kuace3, Kuclr2, Kuace2, or Kuxyr1 was not obviously impaired on plates containing lactose. Lactose is considered to be an economically feasible carbon source for inducing cellulase expression in *T*. *reesei* [34–37]. Therefore, we decided to use lactose as the carbon source for all our subsequent investigations in this study.

**Fig. 3:**
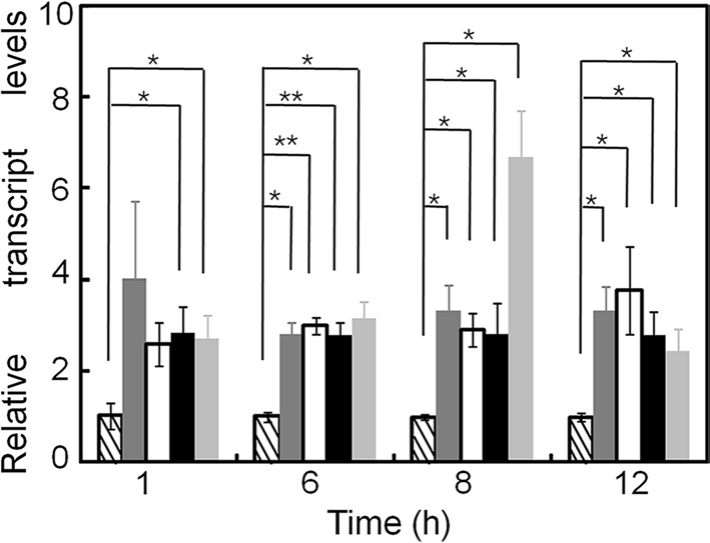
*xyr1* transcriptional levels in the parent strain and fTF-containing transformants. The left slash represents the parent strain, dark gray represents Kuace3, white represents Kuclr2, black represents Kuace2, and light gray represents Kuxyr1. The transcriptional level of *xyr1* was normalized to that of actin (data presented are mean ± SEM; *p < 0.05, **p < 0.01, *n* = 3; two-tailed Student’s *t* tests)

Next, we used qRT-PCR to investigate the expression levels of genes encoding cellulase transcription factors in the Kuace3, Kuclr2, Kuace2, Kuxyr1, and parent strains following their incubation in media containing 2% lactose for 1, 6, 8, and 12 h. The transcriptional levels of most cellulase transcription factors were unaltered (data not shown). However, *xyr1*, which encodes the main cellulase activator in *T*. *reesei* [11], was significantly upregulated in the Kuace3, Kuclr2, Kuace2, and Kuxyr1 strains (Fig. 3).

**Fig. 4:**
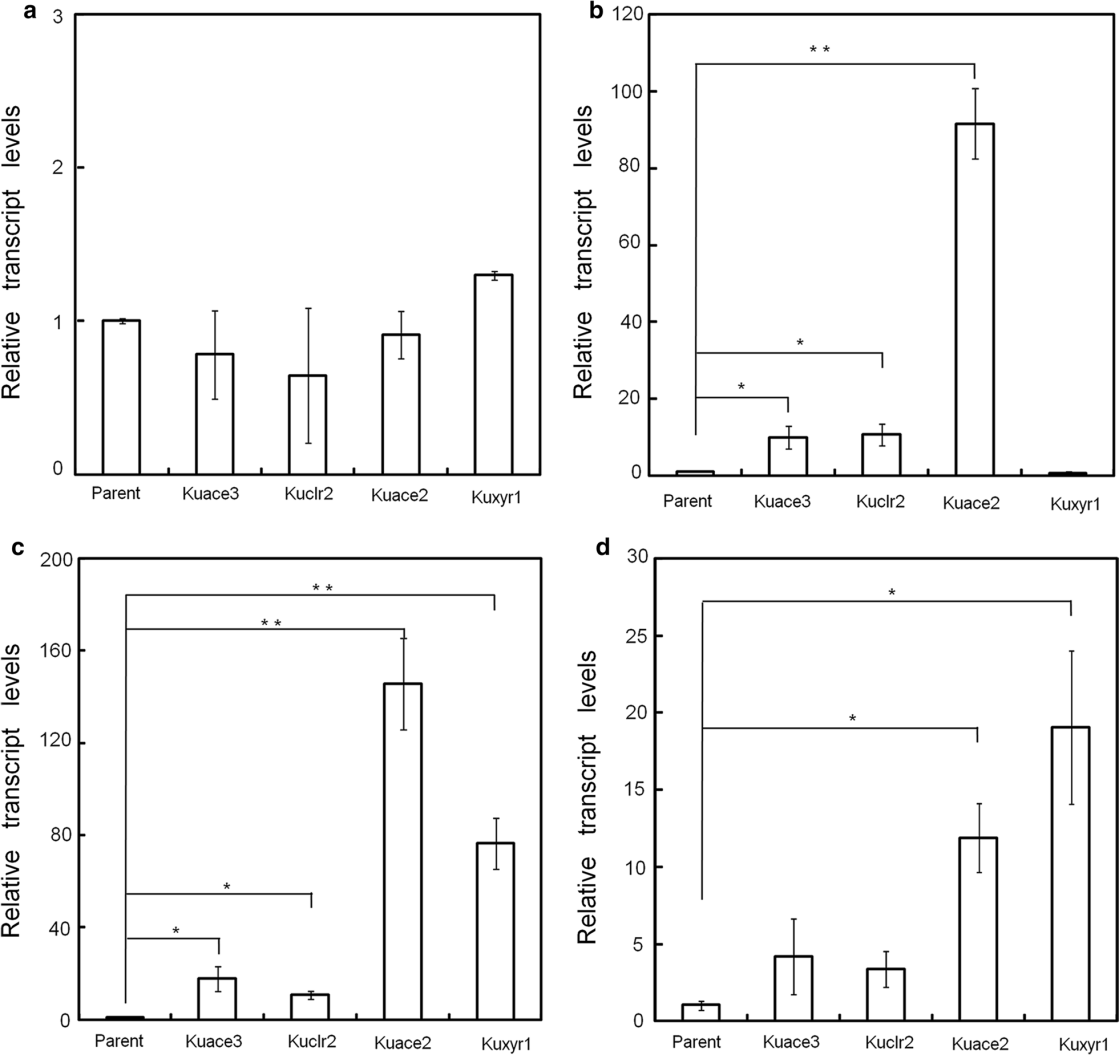
Transcriptional levels of *cel7a* in the parent strain and fTF-containing transformants. **a** Cultivation for 1 h; **b** cultivation for 6 h; **c** cultivation for 8 h; **d** cultivation for 12 h. The transcriptional level of the *cel7a* gene was normalized to that of actin (data presented are mean ± SEM; *p < 0.05, **p < 0.01, ***p < 0.001, *n* = 3; two-tailed Student’s *t* tests)

When *T*. *reesei* Rut-C30 was cultured in a two-stage system with lactose as a carbon source and a controlled pH of 4.8, the major secreted cellulase was cellobiohydrolase 1, which can account for approximately 50% of the total extracellular protein [38]. Cellobiohydrolase 1 is encoded by the *cel7a* gene. Therefore, we examined the transcriptional level of *cel7a* in all our strains. As shown in Fig. 4, the transcriptional levels of *cel7a* in Kuace3, Kuclr2, Kuace2, and Kuxyr1 were all significantly upregulated at 6 (Fig. 4b), 8 (Fig. 4c), and 12 h (Fig. 4d) after transferring to media containing lactose. The expression of *cel7a* in the Kuace2 strain was upregulated by approximately 100-fold at 6 or 8 h following its transfer to lactose-containing media. Furthermore, the expression of *cel7a* was upregulated by approximately 77-fold in the Kuxyr1 strain at 8 h and approximately tenfold in the Kuace2 and Kuxyr1 strains at 12 h. Although the expression of *cel7a* was significantly enhanced in the Kuace3 or Kuclr2 strains at 6, 8, or 12 h, the extent of upregulation was much lower than that of Kuace2 and Kuxyr1. There were no significant changes in the transcriptional levels of the fTFs (*Sace3*, *Sclr2*, *Sace2*, and *Sxyr1*) among the transformants (Additional file 6: Figure S6). Conclusively, although all fTFs were able to significantly improve the transcription of both *xyr1* and *cel7a* in *T*. *reesei*, the augmentations exhibited by strains containing *Sace2* or *Sxyr1* were much higher than those containing *Sace3* or *Sclr2*. Therefore, we concluded that the fTFs must have different functions that led to these observed changes in expression levels.

### Determination of cellulase activity in shake-flask cultures

**Fig. 5:**
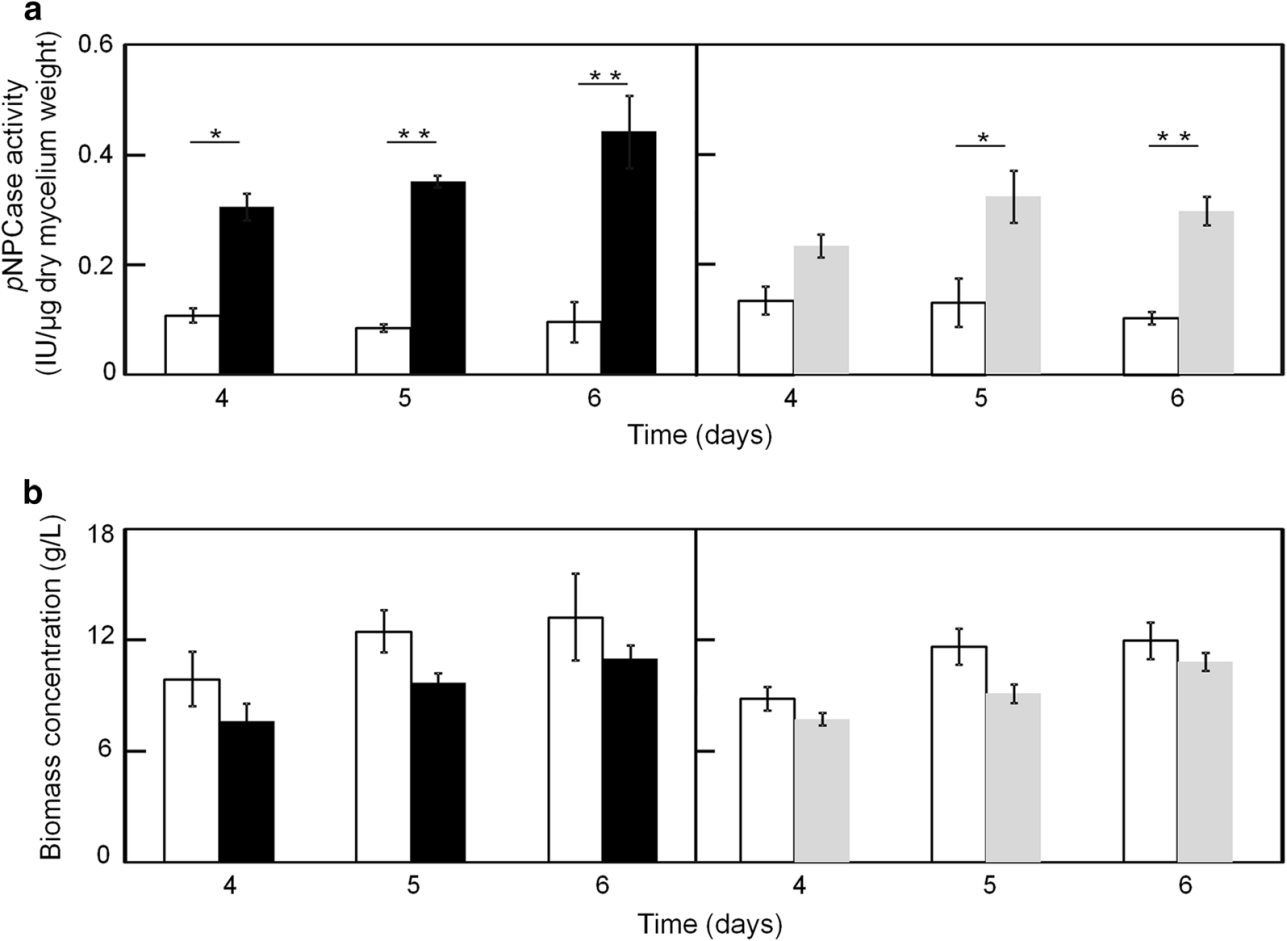
Comparison of *p*NPCase activity and biomass concentration among strains in shake-flask cultures. The white symbol represents the parent strain, black represents Kuace2, and gray represents Kuxyr1. **a** *p*NPCase activity; **b** biomass concentration (data presented are mean ± SEM; *p < 0.05, **p < 0.01, *n* = 3; two-tailed Student’s *t* tests)

As Kuace2 and Kuxyr1 exhibited the highest *cel7a* transcription levels (Fig. 4), we compared the *p*NPCase (cellobiohydrolase 1) activities of Kuace2, Kuxyr1, and the parent strain. For each of these strains, *p*NPCase activities were determined, and the results were expressed as enzyme unit per dry mycelium weight. Although the *p*NPCase activities in Kuace2 and Kuxyr1 were significantly increased compared with that of the parent strain (*p *< 0.05, *n* = 3) (Fig. 5a), the biomass accumulations of these strains were not significantly altered (Fig. 5b). These findings indicate that both *Sace2* and *Sxyr1* are able to significantly enhance *p*NPCase activities in *T*. *reesei* without affecting biomass accumulation.

### Cellulase production in 3-L fermenters

As cellobiohydrolase 1 expression in the Kuace2 or Kuxyr1 strains was significantly elevated in shake-flask cultures (Figs. 4 and 5), we scaled up the fed-batch cultures to assess cellobiohydrolase 1 production by the Kuace2, Kuxyr1, *Δcre1*, and parent strains in 3-L fermenters. Supplementary medium containing lactose as a carbon source and (NH_4_)_2_SO_4_ as a nitrogen source was added after 66 h of cultivation. The intracellular protein concentration was measured to determine the biomass concentration in each culture [39, 40]. In addition, the pH was controlled at 4.0–5.0, and no significant changes in pH values were observed during the whole fermentation processes for any of the strains (data not shown).

**Fig. 6:**
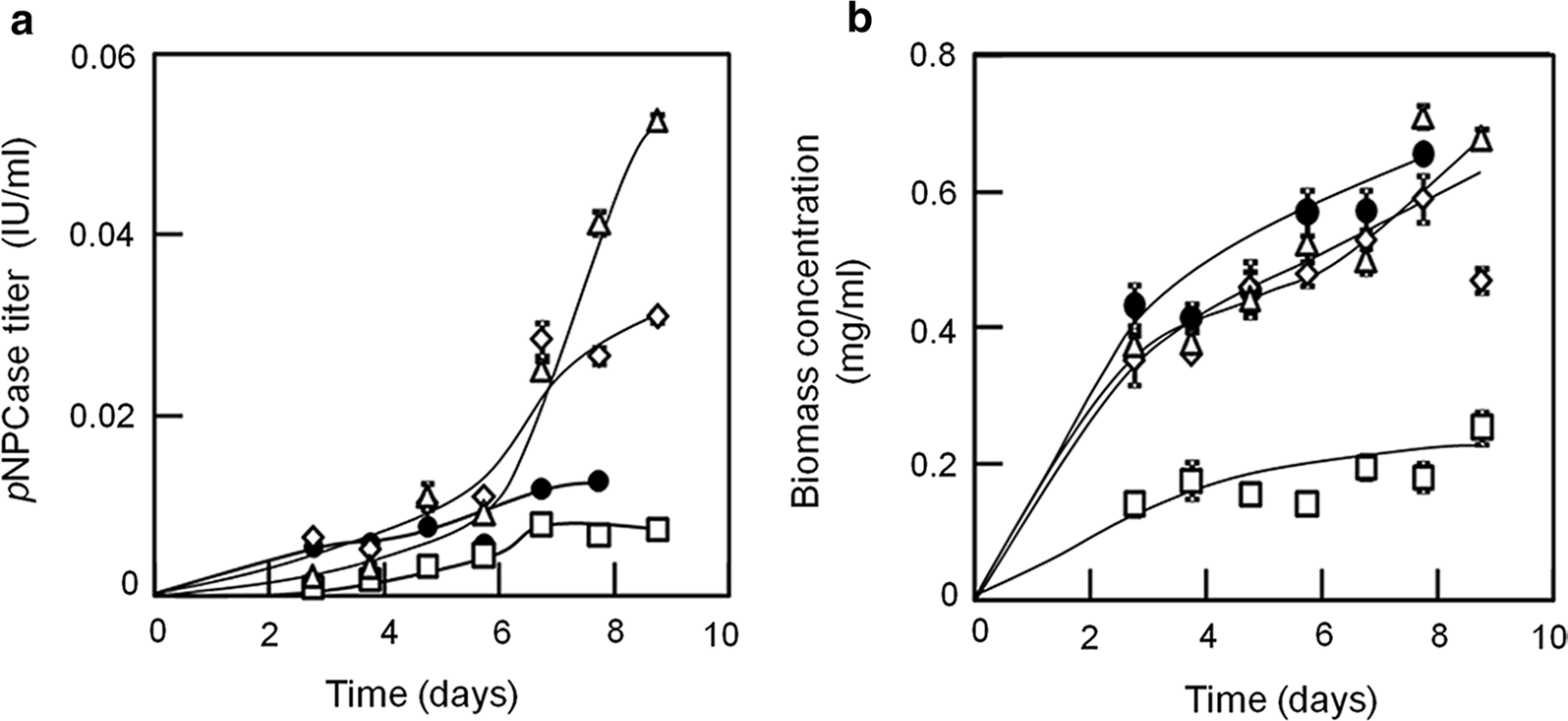
Comparison of *p*NPCase titer and biomass accumulation among strains. The black sphere represents the parent strain, a white triangle represents Kuace2, a white diamond represents Kuxyr1, and a white square represents *Δcre1*. **a** *p*NPCase titer; **b** biomass concentration

As shown in Fig. 6, the *p*NPCase activities were significantly increased in the Kuace2 and Kuxyr1 strains compared with those in the parent and *Δcre1* strains after feeding was complete (*p *< 0.05, *n* = 3). The biomass accumulation of the Kuace2, Kuxyr1, and parent strains significantly increased after 108 h of cultivation and was noted to be maximum at 180 h. In contrast, the biomass concentration of the *Δcre1* strain slowly increased throughout its cultivation (Fig. 6b). We observed no significant differences in biomass accumulation among the Kuace2, Kuxyr1, and parent strains. However, the *Δcre1* strain biomass was approximately one-quarter or one-third of the concentration reached by the Kuace2 or Kuxyr1 strains, respectively. Furthermore, the *p*NPCase titers of Kuace2 and Kuxyr1 were strongly increased following the completion of feeding compared with those of the parent and *Δcre1* strains (Fig. 6a). After 180 h of fermentation, the *p*NPCase titers of Kuace2 and Kuxyr1 were 3.2- and 2.1-fold higher, respectively, than that of the parent strain and 6.1- and 3.9-fold higher, respectively, than that of *Δcre1* strain. To summarize, these results indicated that Kuace2 and Kuxyr1 produced much higher *p*NPCase titers than the parent and *Δcre1* strains, and maintained a similar growth profile to that of the parent strain.

## Discussion

Designing new strategies is important for the production of protein or other biochemicals in fungal species. Recent studies to increase cellulase production have primarily focused on the manipulation of Cre1/Mig1 (a yeast Cre1 homolog), for example, the use of gene knockouts or replacement of the gene with a non-functional allele [11, 20, 41, 42]. However, this can affect biomass accumulation, which results in a decreased final product titer. Lactose is an economically feasible soluble carbon source for inducing cellulase production in *T. reesei* [34–37], which has been widely used in the enzyme industry [43–45]. However, an extracellular β-galactosidase (*bga1*) produced by *T*. *reesei* hydrolyzes lactose into d-galactose and d-glucose, and *bga1* overexpression abolishes the expression of the two prominent cellulase-encoding genes *cbh1* and *cbh2* in *T*. *reesei* [46]. Therefore, CCR can be triggered when using lactose as a carbon source. In addition to *cel7a*, we also observed that the expression of the cellulase genes such as *cel6a*, *cel5a*, and *cel7b* in *T*. *reesei* was significantly elevated in all fTF-containing transformants cultured on plates containing lactose [38] (Additional file 7: Figure S7). Recent studies have reported that an increased cellulolytic activity occurred as a result of the introduction of a truncated form of *cre1*, *cre1*-*96*, into *T*. *reesei* [19, 20]. Although this approach is different from the one described in this study, the overexpression of either *cre1*-*96* or fTF has no significant effect on *T*. *reesei* growth and exerts a positive effect on cellulase production by attenuating the effect of CCR.

Xyr1 is a major activator of cellulase production in *T*. *reesei* [32, 47]. However, lactose-induced *xyr1* expression requires the presence of Cre1 [48]. Therefore, despite mediating CCR, Cre1 also exerts positive effects on cellulase transcription under inducing conditions. Genome-wide analysis has revealed that Cre1 participates in several morphological events in *T*. *reesei*, such as hyphal development and sporulation [18]. The deletion of another *T*. *reesei* cellulase repressor, Ace1, also severely impairs fungal growth [39]. Therefore, the deletion of *cre1* or *ace1* is not a good strategy to improve cellulase production in *T*. *reesei*.

**Fig. 7:**
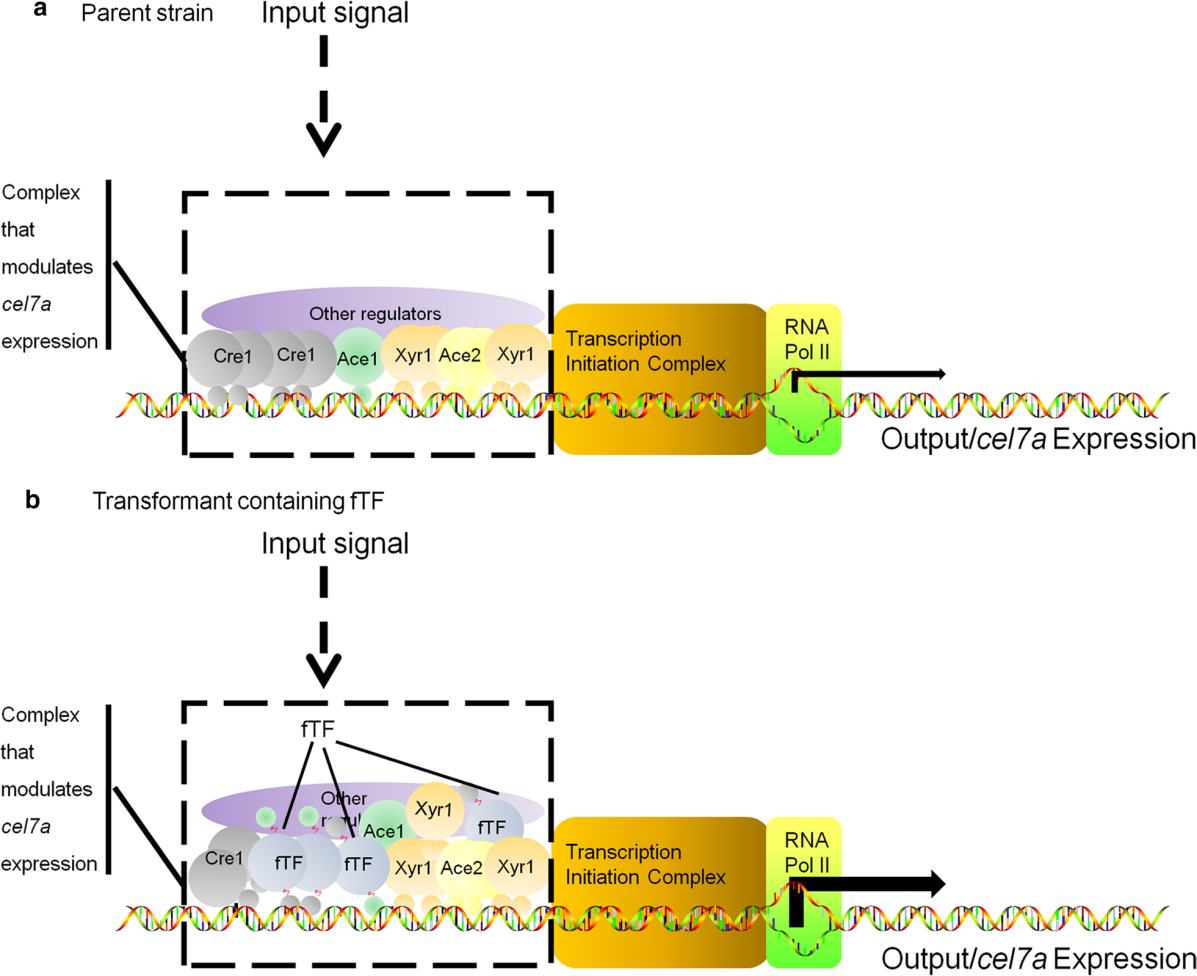
Schematic diagrams showing *cel7a* transcriptional regulation in the parent and transformant strains. **a** parent strain; **b** transformants (Kuace3, Kuclr2, Kuace2, or Kuxyr1)

The *T*. *reesei cel7a* gene has been proposed to be repressed via a Cre1 and Ace1-mediated double lock mechanism: Cre1 or Ace1 directly binds to the promoter region of *cel7a*, followed by the repression of Xyr1 expression by Cre1 and Ace1 [11, 32, 39, 47–49]. Therefore, one possible reason for the fTF-mediated elevation of *cel7a* expression is that fTFs can disturb the Cre1-and Ace1-mediated double lock by competing for Cre1- and Ace1-binding sites after the initiation of CCR during cellulase production. This hypothetical working mechanism is illustrated in Fig. 7.

Although the four fTFs possess the same zinc finger motifs, the *cel7a* transcription level was higher in strains containing *Sace2* or *Sxyr1* than in those containing *Sace3* or *Sclr2*. The possible reasons for this phenomenon include the following: (i) a heterodimer composed of Xyr1 and Ace2 has been demonstrated to function as an activator of cellulase expression; (ii) a Xyr1 homodimer has been implicated in the formation of a cellulase induction-specific complex [50, 51]; (iii) there is no report on a homodimer or heterodimer composed of Ace3 or Clr2 found in the cellulase promoter regions in *T*. *reesei*; and (iv) it is possible that *Sace2* and *Sxyr1* may form more hetero- or homodimers with Xyr1 at the *cel7a* promoter region than with *Sace3* or *Sclr2*. Future studies will involve the introduction of the four fTFs (*Sace3*, *Sclr2*, *Sace2*, and *Sxyr1*) into *T. reesei Δxyr1* (*tku70*::*hph*; *xyr1*::*pyr4*) or *T. reesei Δace2* (*tku70*::*hph*; *ace2*::*pyr4*). The transcriptional levels of cellulase genes will be compared among all the transformants to investigate the relationship between fTFs and homodimer/heterodimer formation. These will be helpful for further understanding the role of the Xyr1 and Ace2 activation domains on cellulase transcription.

Synthetic or fusion transcription factors have been prepared for exploring the transcriptional regulation network and phenotypic engineering in yeast and mammalian cells [21, 22]. However, these studies do not address the delicate balance between product titer and strain growth. In this study, we designed four fusion or synthetic transcription factors in *T*. *reesei*, and found that two fTFs, *Sace2* and *Sxyr1*, significantly improve the expression of a major cellulase gene without decreasing biomass accumulation and resulted in significantly enhanced cellulase activities. It is possible that the strategies described in this study could be applied in other fungal species, as phenomena related to Cre1-mediated CCR have been observed. For example, in the oleaginous yeast *Yarrowia lipolytica*, the growth rate and biomass accumulation of *Δmig1* (a homologous gene of *cre1*) were found to be lower than those of the wild type strain; however, a 1.35-fold lipid increase in lipid content was observed [41].

## Conclusions

Here, we developed a new strategy for increasing *T*. *reesei* cellulase titers. Four fTFs (*Sace3*, *Sclr2*, *Sace2*, and *Sxyr1*) were designed to release or attenuate the CCR inhibition of cellulase genes, and Cre1 was left intact to maintain strain growth. The Kuace3, Kuclr2, Kuace2, and Kuxyr1 strains were produced by transforming *Sace3*, *Sclr2*, *Sace2*, and *Sxyr1*, respectively, into the *T*. *reesei* genome. The growth of these transformants was roughly equivalent to that of the parent strain. However, the transcription levels of *cel7a*, which encodes the major cellulase protein cellobiohydrolase 1, were significantly elevated in all transformants, particularly those in Kuace2 and Kuxyr1, when lactose was used as the carbon source. This suggests that a complete or partial release from CCR occurred. Furthermore, the use of fed-batch fermentation resulted in a significant increase in the *p*NPCase titers of the Kuace2 or Kuxyr1 strains compared with those of the parent and *Δcre1* strains.

To summarize, a new strategy based on fTFs was successfully established to improve cellulase titers in *T*. *reesei*. In addition, this study provides valuable information for designing new fTFs for biochemical compounds production in other fungal species.

## Methods

### Strains and reagents

*Trichoderma reesei Δtku70* (ATCC MYA-256), a nonhomologous end joining pathway-deficient strain, was used as the parent strain in this study [52]. All the other strains constructed in this study are listed in Additional file 8: Table S1. The *E. coli* strain GB05-dir was used for constructing all the plasmids [53].

Wheat bran was kindly provided by Longlive Bio-Technology Co., Ltd. (Yucheng, Shandong, China). The *p*-nitrophenyl-β-d-cellobioside (*p*NPC) was purchased from Sigma-Aldrich (St. Louis, MO, USA). Uridine, PEG6000, sorbitol, and lactose were purchased from Sangon Biotech Co., Ltd. (Shanghai, China). KOD FX DNA polymerase (Toyobo Co., Ltd., Osaka, Japan) was used for all polymerase chain reaction amplifications. RNAiso™ reagent, PrimeScript^®^ RT reagent Kit With gDNA Eraser (Perfect Real Time) and SYBR^®^ Premix Ex Tag™ (Tli RNase H Plus) were purchased from Takara Bio Inc. (Shiga, Japan). The DIG High Prime DNA Labeling and Detection Starter Kit I (Roche Diagnostics, Mannheim, Germany) was used for Southern blotting analysis. The restriction enzymes *Ase* I and *Xho* I used for genome digestion were purchased from Thermo Fisher Scientific Inc. (Waltham, MA, USA). All other chemicals were purchased from Sinopharm Chemical Reagent Co., Ltd. (Shanghai, China).

### Construction of fTF replacement cassettes and the *cre1* deletion cassette

All plasmids were constructed by linear–linear homologous recombination, which was mediated by full-length RecET in *E. coli* GB05-dir [53, 54]. The construction and composition of the pSxyr1 plasmid containing the *Sxyr1* replacement cassette are as follows: 2 kb of sequence up- and downstream of the *ku70* gene, the promoter and zinc finger regions of *cre1* (comprising nucleotides − 1364 to 417 bp), the activation domain of *xyr1* (445 to 2947 bp), and the zinc finger region of *ace1* (1276 to 1716 bp) were each amplified from the *T*. *reesei* genome. A GSGGSGTS peptide was then used to link each domain. The *pyr4* selective marker was obtained as previously described [55], and the *trpC* terminator was cloned from pSilent-1. The pUG6 plasmid was used as the accepter plasmid. A similar strategy was used to construct the pSace3, pSclr2, and pSace2 plasmids. The activation domains of *ace3* (comprising nucleotides 220–2035 bp), *clr2* (100–2003 bp), and *ace2* (118–1023 bp) were inserted into pSace3, pSclr2, and pSace2, respectively. A schematic diagram of the *Sace3*, *Sclr2*, *Sace2*, and *Sxyr1* replacement cassettes is shown in Additional file 1: Figure S1A. The composition of the pCre1 plasmid containing the *cre1* deletion cassette included the 2 kb up- and downstream of the *ku70* gene, the *pyr4* selective marker, and the pUG6 plasmid. A schematic diagram of the *cre1* deletion cassette is shown in Additional file 2: Figure S2A.

### Strain construction

The transformation of *Sace3*, *Sclr2*, *Sace2*, and *Sxyr1* replacement cassettes and the *cre1* deletion cassette into *T*. *reesei Δtku70* was performed using previously described methods [52]. The transformants were cultured on plates containing minimal medium (MM) + 2% glucose. The composition of MM is described in the literature [55]. Following two rounds of single-spore isolations, correct transformants with homologous integration were confirmed using PCR and Southern blotting.

### Growth and sporulation assays

Approximately 1 cm^3^ of agar containing parent or mutant mycelia was inoculated on a solid plate containing PDA, wheat bran, MM with 2% glucose, or MM with 2% lactose. All media used for *T*. *reesei Δtku70* culture were supplemented with 0.001% uridine. Plates were incubated at 30 °C for 6 days, and colony diameters were measured from the 2nd day. All experiments were performed in triplicates. Microscopic observation of hyphal morphology was based on a previously described method [56]. Approximately, 1 × 10^3^ spores were inoculated on coverslips with solidified medium containing PDA, wheat bran, MM with 2% glucose, or MM with 2% lactose at 30 °C for 48 h. Microscopic images of hyphae (Nikon Eclipse E100, 400× magnification) were then captured using a Nikon D5000 camera.

Approximately, 1 × 10^6^ spores of parent strain and transformants were inoculated on PDA and wheat bran plates and incubated at 30 °C for 6 days. Spores were then harvested from the plates using a solution containing 0.9% NaCl and 0.5% Tween-80 and enumerated with a hemocytometer. Colony diameters were measured on the 6th day of incubation. The number of spores produced was normalized to the colony area.

### Identification of fTFs that improve cellulase transcription in *T*. *reesei*

Approximately, 1 × 10^7^ spores of the parent strain and transformants containing fTFs were inoculated into liquid medium containing MM + 2% glucose + 0.001% tryptone and cultured at 30 °C and 200 rpm for 48 h. The mycelia were filtered and washed with MM solution. Equal amounts of mycelia were transferred into submerged medium containing MM + 105 mM glycerol and the samples were incubated at 30 °C and 200 rpm for 24 h. The mycelia were harvested, and approximately 0.1 g of harvested mycelia was transferred to 100 mL liquid medium containing MM + 2 g lactose. The mycelia were cultured at 30 °C and 200 rpm. Samples were taken at 1, 6, 8, and 12 h after inoculation and immediately frozen at − 80 °C. These samples were used for determining the transcriptional levels of cellulase transcription factors and cellulase genes.

The extraction of total RNA and subsequent synthesis of cDNA were performed as previously described [57]. Real-time PCR reactions were performed using a LightCycler 96 Real-Time PCR system (Roche Applied Science, Mannheim, Germany). The real-time PCR reactions were performed according to the manufacturer’s instructions for SYBR Premix Ex Taq™ (Ti RNaseH Plus) (Takara Bio Inc., Shiga, Japan). The relative transcription levels of cellulase genes and transcription factors were calculated using the 2^−ΔΔCT^ method [58]. The primers used for real-time PCR are listed in Additional file 9: Table S2.

### Assessment of *p*NPCase activity

To determine *p*NPCase activities, mycelia were precultured as described above. Approximately 0.2 g of mycelia was inoculated in 200 mL submerged medium containing MM solution + 4 g lactose and incubated at 30 °C and 200 rpm. Culture samples (30 mL) were collected on days 4, 5, and 6 after inoculation and centrifuged at 12,000 rpm for 10 min. The solid sediment was extracted and dried at 100 °C for 24 h [39]. The supernatant (25 mL) was extracted and concentrated to a final volume of 1 mL using Vivaspin 15R centrifugal devices with a 3 kDa molecular weight cutoff (Sartorius, Goettingen, Germany). The concentrated supernatant was then mixed with 20 mL of 50 mM acetic acid buffer (pH 4.8) and concentrated again to a final volume of 1 mL. The *p*NPCase activity was measured as previously described [55] and normalized using the dry mycelium weight.

### Cellulase production using a fed-batch culture in a 3-L fermenter

*Trichoderma reesei* was cultured using a protocol based on a previously published method [43]. Briefly, samples of the parent and mutant strains (approximately 1 × 10^7^ spores) were inoculated in 100 mL liquid medium containing MM solution + 2% glucose + 0.001% tryptone and incubated at 30 °C and 200 rpm. After 48 h of cultivation, the samples were used as seed cultures for fermentation. The seed culture (200 mL) was used to inoculate a liquid medium containing 1.8 L of MM solution, 6 g glucose, 10 g Avicel, and 2 g tryptone in a 3-L fermenter (Model BLB10-3GJG, Bailun Bio-Technology Co., Ltd., Shanghai, China). The feeding solutions contained 400 mL of carbon source (150 g lactose/L) and 400 mL of nitrogen source (160 g (NH_4_)_2_SO_4_/L). The initial pH value was regulated at pH 4.5 and maintained at pH 4.0–5.0 by the automatic addition of 3 M H_3_PO_4_ and 3 M KOH during the entire fermentation process. The airflow was 1.5 L/min and the agitation rate was adjusted to maintain the dissolved oxygen level above 30%. The media were fed following 66 h of cultivation. The injection rates of the carbon and nitrogen solutions were 0.72 g/h and 0.23 g/h, respectively. Samples were periodically collected to measure the *p*NPCase titer and intracellular protein concentration. The measurements of intracellular protein concentration to determine the biomass concentration were performed according to a previously described method [56].

### Statistical analysis

All experiments were performed in triplicates. Statistical analyses were performed using two-tailed Student’s *t* tests, and *p* values of < 0.05 were considered statistically significant.

## Supplementary information

**Additional file 1: Figure S1.** Southern blotting analysis of parent and fTF-containing transformants. A) Schematic diagram of Southern blotting. B) Southern blot of the parent, Kuace3, and Kuclr2 strains. C) Southern blot of the parent, Kuace2, and Kuxyr1 strains. 1: 2 kb upstream of *ku70*; 2: *cre1* promoter; 3: *Sace3*; 4: *trpC* terminator; 5: *pyr4*; 6: 2 kb downstream of *ku70*; 7: *Sclr2*; 8: *Sace2*; 9: *Sxyr1*. Lane M: 1 kb molecular weight marker, Lane 1: parent strain, Lane 2: Kuace3 strain, Lane 3: Kuclr2 strain, Lane 4: Kuace2 strain, Lane 5: Kuxyr1 strain. The arrows indicate the predicted size of each band.

**Additional file 2: Figure S2.** Southern blotting analysis of the *Δcre1* strain. A) Schematic diagram of Southern blotting. B) Southern blot of parent and *Δcre1* strains. 1: 2 kb upstream of *cre1* gene; 2: *pyr4*; 3: 2 kb downstream of *cre1*. Lane M: 1 kb molecular weight marker, Lane 1: parent strain, Lane 2: *Δcre1* strain. The arrows indicate the predicted size of each strain.

**Additional file 3: Figure S3.** Colony diameters of parent strain and transformants on plates containing different carbon sources. Black represents cultivation for 2 days, white represents cultivation for 3 days, dark gray represents cultivation for 4 days, and light gray represents cultivation for 5 days.

**Additional file 4: Figure S4.** Microscopic observation of the hyphae of the parent strain and transformants. Scale: 100 μm.

**Additional file 5: Figure S5.** Sporulation of the parent strain and transformants. Black represents wheat bran as the culture medium; white represents PDA as the culture medium.

**Additional file 6: Figure S6.** Transcriptional levels of fTFs among the transformants. The transcriptional level of fTFs was normalized to that of actin. Dark gray represents Kuace3, white represents Kuclr2, black represents Kuace2, and light gray represents Kuxyr1.

**Additional file 7: Figure S7.** Transcriptional levels of *cel6a*, *cel5a*, and *cel7b* among the transformants. The transcriptional levels of *cel6a*, *cel5a*, and *cel7b* were normalized to that of actin (data presented are mean ± SEM; * *p *< 0.05, ** *p *< 0.01, *** *p* < 0.001, n = 3; two-tailed Student’s *t* tests). A left slash represents the parent strain, dark gray represents Kuace3, white represents Kuclr2, black represents Kuace2, and light gray represents Kuxyr1.

**Additional file 8: Table S1.** *T. reesei* strains used in this study.

**Additional file 9: Table S2.** Primers used for real-time PCR.

## Data Availability

All data generated or analyzed during this study are included in this published article and the additional files.

## Abbreviations

CCR: Carbon catabolite repression
fTF: Fusion transcription factor
*p*NPC: *p*-Nitrophenyl-β-d-cellobioside
MM: Minimal medium
PDA: Potato dextrose agar
