# Supplementary figures and images for: Use of fusion transcription factors to reprogram cellulase transcription and enable efficient cellulase production in Trichoderma reesei
Source: Biotechnol Biofuels. 2019 Oct 15;12:244. doi: 10.1186/s13068-019-1589-2 (PMC6792246; doi:10.1186/s13068-019-1589-2)

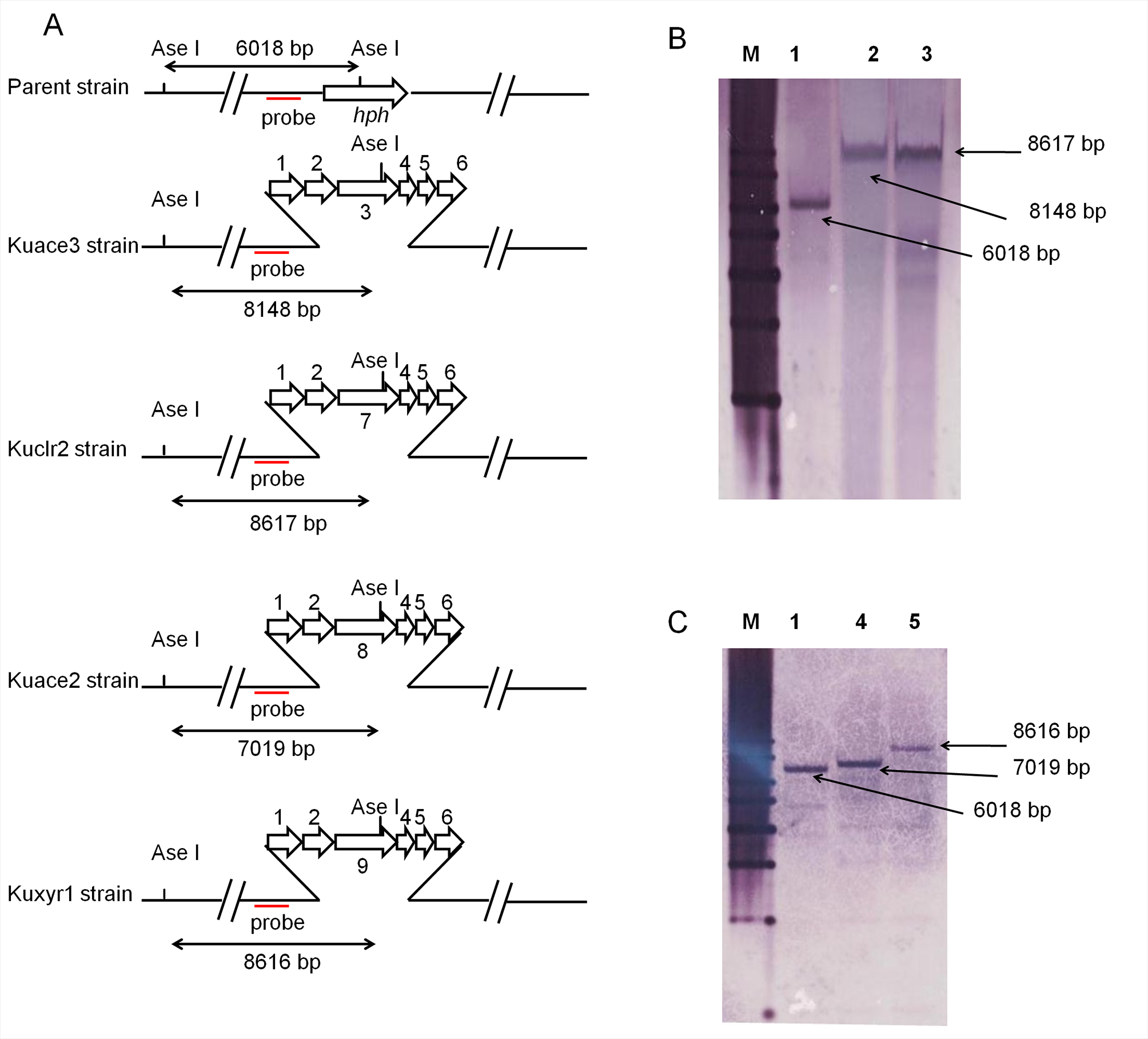

Supplement: Supplementary file 1 — Additional file 1: Figure S1. Southern blotting analysis of parent and fTF-containing transformants. A) Schematic diagram of Southern blotting. B) Southern blot of the parent, Kuace3, and Kuclr2 strains. C) Southern blot of the parent, Kuace2, and Kuxyr1 strains. 1: 2 kb upstream of ku70; 2: cre1 promoter; 3: Sace3; 4: trpC terminator; 5: pyr4; 6: 2 kb downstream of ku70; 7: Sclr2; 8: Sace2; 9: Sxyr1. Lane M: 1 kb molecular weight marker, Lane 1: parent strain, Lane 2: Kuace3 strain, Lane 3: Kuclr2 strain, Lane 4: Kuace2 strain, Lane 5: Kuxyr1 strain. The arrows indicate the predicted size of each band. [file 13068_2019_1589_MOESM1_ESM.tif]

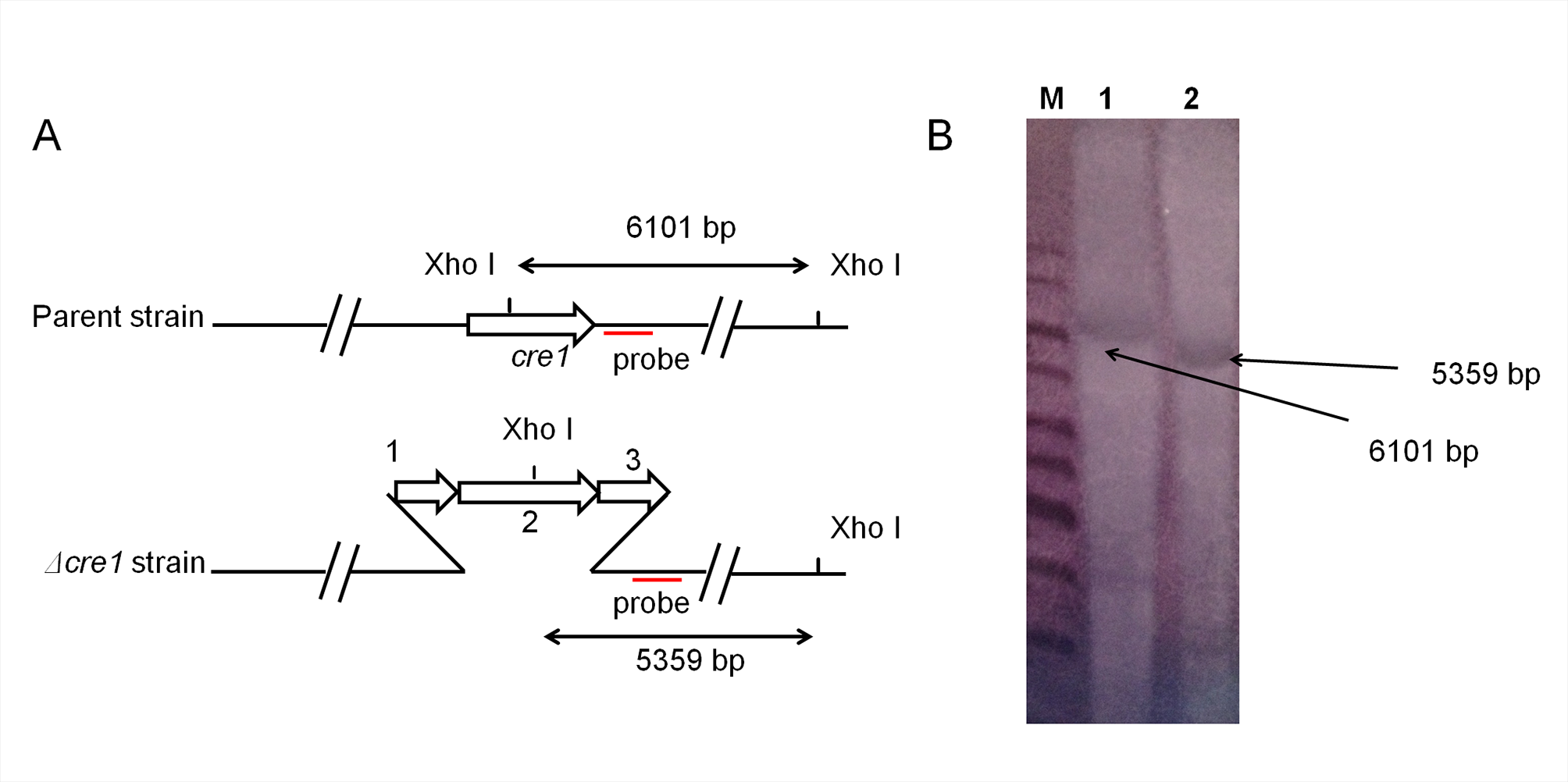

Supplement: Supplementary file 2 — Additional file 2: Figure S2. Southern blotting analysis of the Δcre1 strain. A) Schematic diagram of Southern blotting. B) Southern blot of parent and Δcre1 strains. 1: 2 kb upstream of cre1 gene; 2: pyr4; 3: 2 kb downstream of cre1. Lane M: 1 kb molecular weight marker, Lane 1: parent strain, Lane 2: Δcre1 strain. The arrows indicate the predicted size of each strain. [file 13068_2019_1589_MOESM2_ESM.tif]

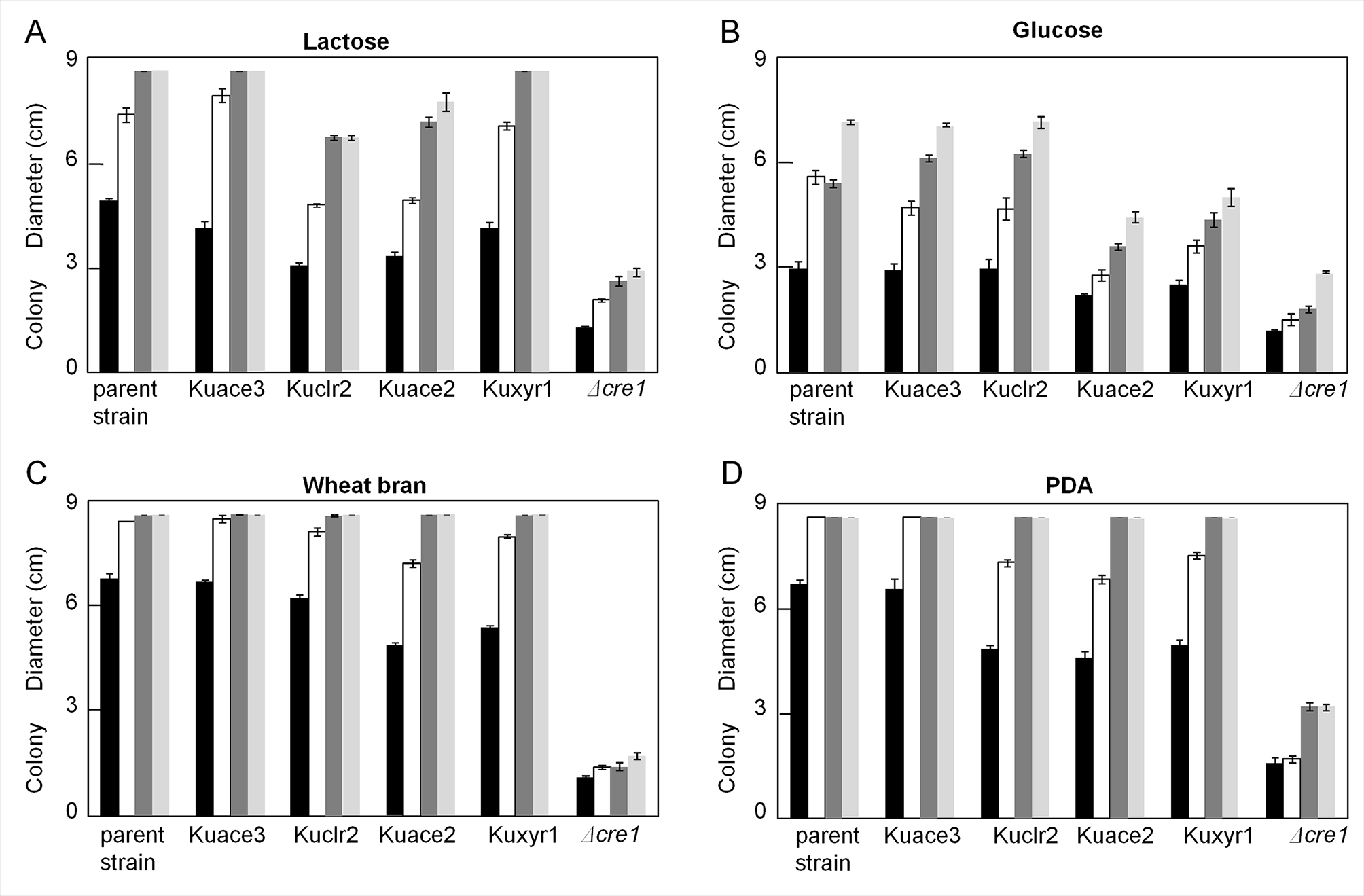

Supplement: Supplementary file 3 — Additional file 3: Figure S3. Colony diameters of parent strain and transformants on plates containing different carbon sources. Black represents cultivation for 2 days, white represents cultivation for 3 days, dark gray represents cultivation for 4 days, and light gray represents cultivation for 5 days. [file 13068_2019_1589_MOESM3_ESM.tif]

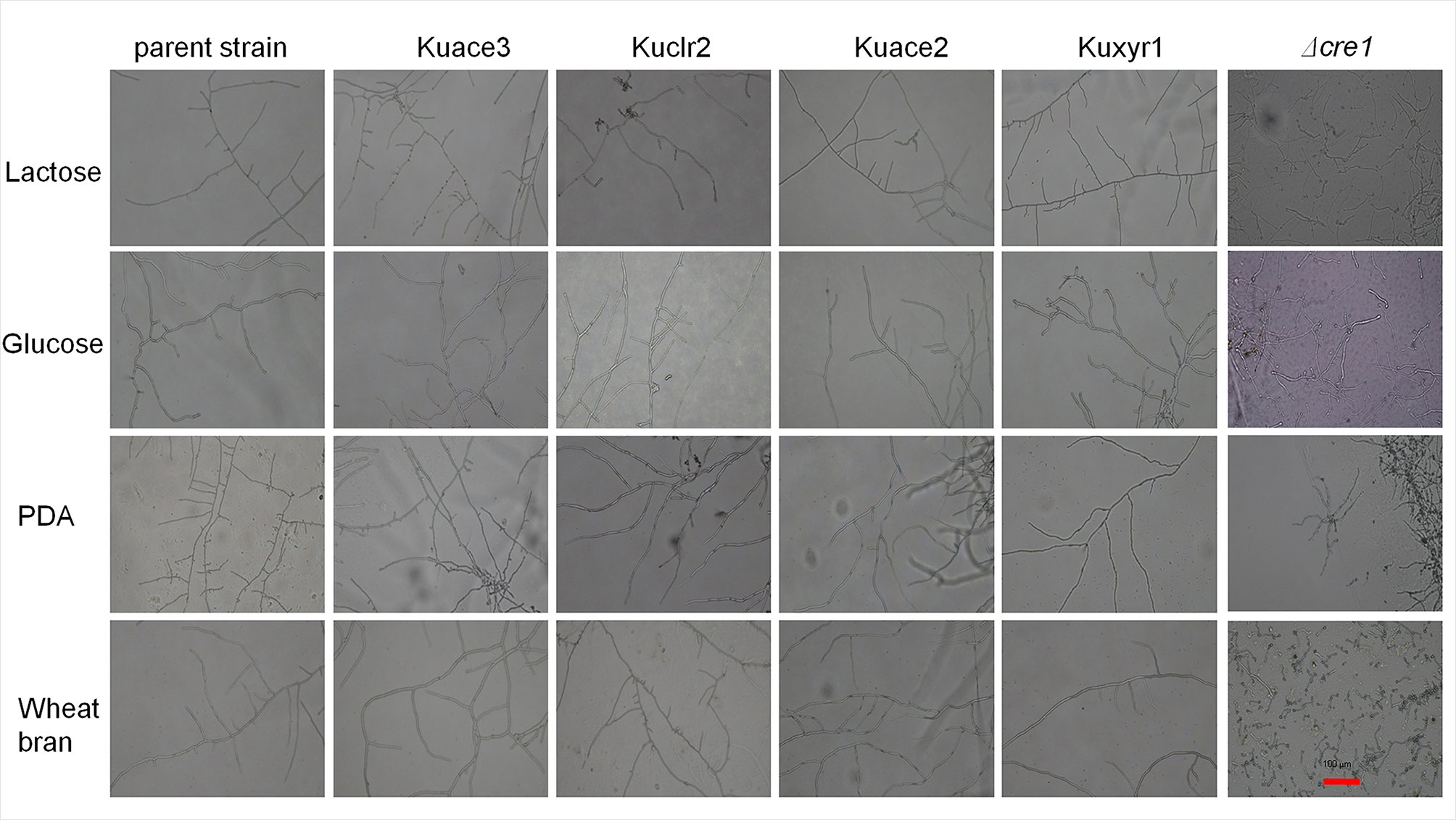

Supplement: Supplementary file 4 — Additional file 4: Figure S4. Microscopic observation of the hyphae of the parent strain and transformants. Scale: 100 μm. [file 13068_2019_1589_MOESM4_ESM.tif]

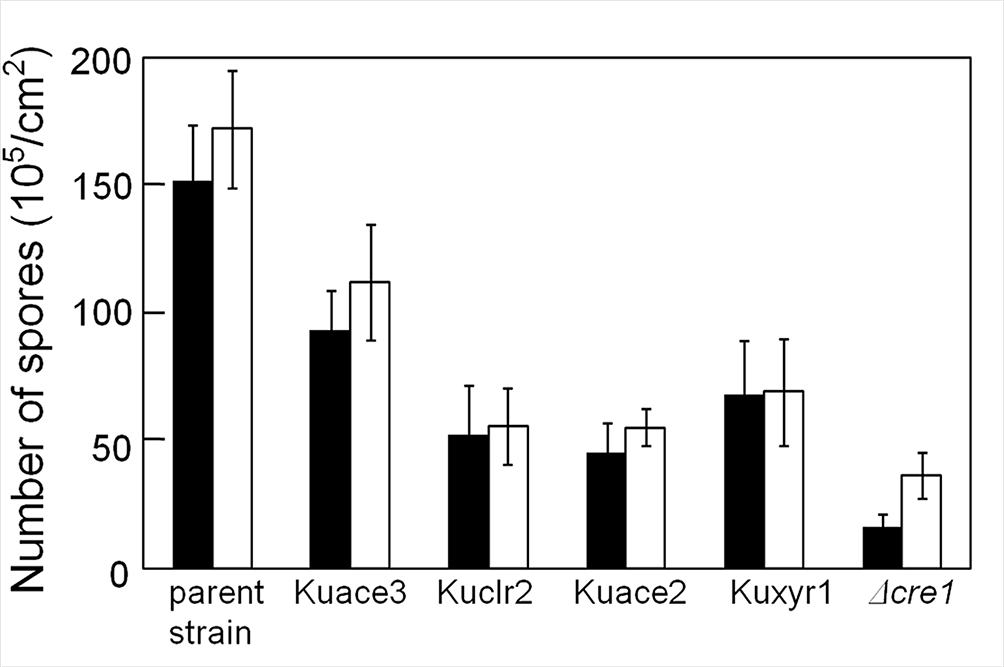

Supplement: Supplementary file 5 — Additional file 5: Figure S5. Sporulation of the parent strain and transformants. Black represents wheat bran as the culture medium; white represents PDA as the culture medium. [file 13068_2019_1589_MOESM5_ESM.tif]

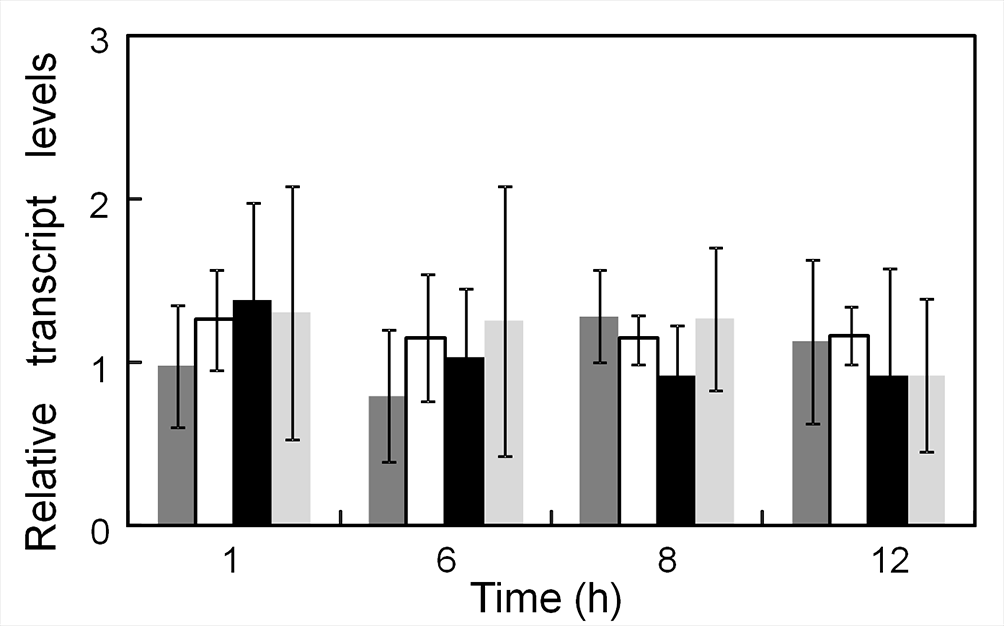

Supplement: Supplementary file 6 — Additional file 6: Figure S6. Transcriptional levels of fTFs among the transformants. The transcriptional level of fTFs was normalized to that of actin. Dark gray represents Kuace3, white represents Kuclr2, black represents Kuace2, and light gray represents Kuxyr1. [file 13068_2019_1589_MOESM6_ESM.tif]

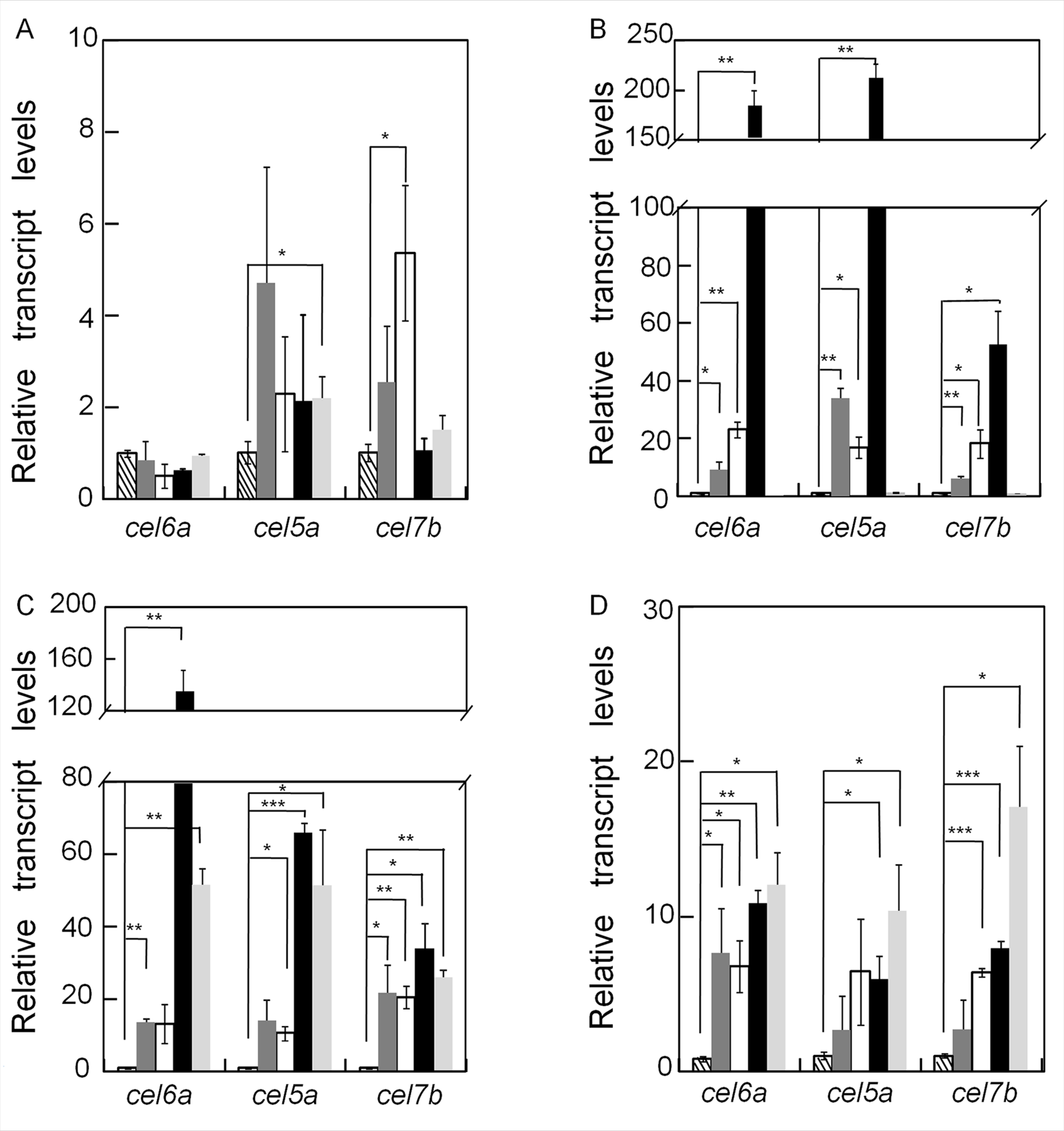

Supplement: Supplementary file 7 — Additional file 7: Figure S7. Transcriptional levels of cel6a, cel5a, and cel7b among the transformants. The transcriptional levels of cel6a, cel5a, and cel7b were normalized to that of actin (data presented are mean ± SEM; * p < 0.05, ** p < 0.01, *** p < 0.001, n = 3; two-tailed Student’s t tests). A left slash represents the parent strain, dark gray represents Kuace3, white represents Kuclr2, black represents Kuace2, and light gray represents Kuxyr1. [file 13068_2019_1589_MOESM7_ESM.tif]
