# Supplementary material for: Use of fusion transcription factors to reprogram cellulase transcription and enable efficient cellulase production in Trichoderma reesei
Source: Biotechnol Biofuels. 2019 Oct 15;12:244. doi: 10.1186/s13068-019-1589-2 (PMC6792246; doi:10.1186/s13068-019-1589-2)
Supplement: Supplementary file 8 — Additional file 8: Table S1. T. reesei strains used in this study. [file 13068_2019_1589_MOESM8_ESM.docx]

Table S1 *T. reesei* was used in this study

| Strains | Genotype | Source |
| --- | --- | --- |
| *T. reesei Δtku70* | *tku70*::*hph*; *Δpyr4* | [1] |
| *T. reesei* Kuace3 | *hph*:: *Sace3* | This study |
| *T. reesei* Kuclr2 | *hph*:: *Sclr2* | This study |
| *T. reesei* Kuace2 | *hph*:: *Sace2* | This study |
| *T. reesei* Kuxyr1 | *hph*:: *Sxyr1* | This study |
| *T. reesei* *Δcre1* | *tku70*::*hph*; *cre1*:: *pyr4* | This study |

Reference

[1] Zhang GT, Hartl L, Schuster A, Polak S, Schmoll M, Wang TH, Seidl V, Seiboth B. Gene targeting in a nonhomologous end joining deficient *Hypocrea jecorina*. *J Biotechnol* 2009;**139**:146-151.
