# Supplementary material for: Use of fusion transcription factors to reprogram cellulase transcription and enable efficient cellulase production in Trichoderma reesei
Source: Biotechnol Biofuels. 2019 Oct 15;12:244. doi: 10.1186/s13068-019-1589-2 (PMC6792246; doi:10.1186/s13068-019-1589-2)
Supplement: Supplementary file 9 — Additional file 9: Table S2. Primers used for real-time PCR. [file 13068_2019_1589_MOESM9_ESM.docx]

Table S2 Primers used for real-time PCR

| Gene | Probe type | Sequence |
| --- | --- | --- |
| *cre1* | Forward | TTTCTTCGTACCACATGGCG |
| *cre1* | Reversed | ACAGGTTTCTCAGACTCGGC |
| *ace1* | Forward | ATCGGCTTTTGATGATGTCG |
| *ace1* | Reversed | CGCTCAGGCTCTTGTTTGTT |
| *xyr1* | Forward | CCTCTCCGTCGCTATTCTGC |
| *xyr1* | Reversed | ATGGGATAGGGGTGGCTGTT |
| *ace2* | Forward | GTGGAGGCGATGGAGG |
| *ace2* | Reversed | CAGAGACCGATGGGAAATG |
| *ace3* | Forward | CGCTACTCCCCCGTCTTA |
| *ace3* | Reversed | GCTGGTCGCTCTTCTTCC |
| *clr2* | Forward | CTTGGCTTTTCCCATTCC |
| *clr2* | Reversed | GCTGCTCATACACGCACC |
| *Sace2* | Forward | CAAGAAGTTCTCCCGCTC |
| *Sace2* | Forward | TGACGCCTACCCCGTTATGAT |
| *Sxyr1* | Forward | AGGATACACAGCAACCCCAAC |
| *Sxyr1* | Reversed | CACGCTTCTTTCTCTCTCGGA |
| *Sace3* | Forward | GCGACTTTTCCGTGCTTTTG |
| *Sace3* | Reversed | GGGGTGGCGTTCTTCTTCCG |
| *Sclr2* | Forward | AATGCCGTGTGGTTTCTGTA |
| *Sclr2* | Reversed | GGGGGTGGCGTTCTTCTTCC |
| *actin* | Forward | TTAAGAAAGCCGCCACCCCC |
| *actin* | Reversed | GTTGGTCGACAGGGAGAGGATG |
| *cel7a* | Forward | CTGCGACTGGAACCCATACC |
| *cel7a* | Reversed | AAGTGACGCCATTCTGGACAT |
| *cel7b* | Forward | CCCGAGGTCCATCCCAAG |
| *cel7b* | Reversed | TCCAGCGGTAGTTCCAGTCAA |
| *cel5a* | Forward | CAACGACGACGGGATGACTA |
| *cel5a* | Reversed | GATGCCAACTGCGACCAAA |
| *cel6a* | Forward | CGTTGCTGGATTCGTTTGTC |
| *cel6a* | Reversed | GATGGGTTTGCGTTTGTGAG |
